# Supplementary material for: Trends in urinary tract infection hospitalization in older adults in Spain from 2000-2015
Source: PLoS One. 2021 Sep 29;16(9):e0257546. doi: 10.1371/journal.pone.0257546 (PMC8480842; doi:10.1371/journal.pone.0257546)
Supplement: S2 File — (DOCX) [file pone.0257546.s002.docx]

Supl 2

|  | **Total UTI** | | | | | | **Non-specified UTI** | | | | | | **Cystitis** | | | | | | **Pyelonephritis** | | | | | | **Prostatitis** | | |
| --- | --- | --- | --- | --- | --- | --- | --- | --- | --- | --- | --- | --- | --- | --- | --- | --- | --- | --- | --- | --- | --- | --- | --- | --- | --- | --- | --- |
|  |  |  |  |  |  |  |  |  |  |  |  |  |  |  |  |  |  |  |  |  |  |  |  |  |  |  |  |
|  | 65-74 | | 75-84 | | >85 | | 65-74 | | 75-84 | | >85 | | 65-74 | | 75-84 | | >85 | | 65-74 | | 75-84 | | >85 | | 65-74 | 75-84 | >85 |
|  | M | W | M | W | M | W | M | W | M | W | M | W | M | W | M | W | M | W | M | W | M | W | M | W | M | M | M |
| **2000** | 1.42 | 1.11 | 2.99 | 2.46 | 6.41 | 4.37 | 0.91 | 0.66 | 2.42 | 1.88 | 5.71 | 3.81 | 0.01 | 0.01 | 0.02 | 0.02 | 0.05 | 0.03 | 0.28 | 0.44 | 0.37 | 0.56 | 0.49 | 0.52 | 0.22 | 0.18 | 0.15 |
| **2001** | 1.44 | 1.16 | 3.13 | 2.58 | 7.02 | 5.00 | 0.94 | 0.71 | 2.55 | 2.01 | 6.23 | 4.50 | 0.01 | 0.01 | 0.03 | 0.02 | 0.04 | 0.02 | 0.26 | 0.45 | 0.40 | 0.55 | 0.56 | 0.48 | 0.22 | 0.16 | 0.20 |
| **2002** | 1.42 | 1.15 | 3.05 | 2.45 | 6.61 | 4.86 | 0.93 | 0.67 | 2.45 | 1.88 | 5.91 | 4.30 | 0.01 | 0.01 | 0.02 | 0.02 | 0.03 | 0.02 | 0.27 | 0.47 | 0.38 | 0.56 | 0.50 | 0.54 | 0.21 | 0.19 | 0.18 |
| **2003** | 1.48 | 1.20 | 3.24 | 2.85 | 7.31 | 5.94 | 0.98 | 0.74 | 2.64 | 2.28 | 6.65 | 5.37 | 0.01 | 0.01 | 0.02 | 0.02 | 0.05 | 0.03 | 0.27 | 0.46 | 0.34 | 0.55 | 0.47 | 0.54 | 0.22 | 0.24 | 0.14 |
| **2004** | 1.56 | 1.31 | 3.26 | 2.87 | 7.46 | 5.63 | 1.05 | 0.80 | 2.63 | 2.25 | 6.75 | 5.05 | 0.01 | 0.01 | 0.02 | 0.02 | 0.06 | 0.03 | 0.28 | 0.50 | 0.38 | 0.60 | 0.50 | 0.55 | 0.22 | 0.23 | 0.14 |
| **2005** | 1.63 | 1.26 | 3.44 | 2.88 | 7.47 | 6.23 | 1.04 | 0.78 | 2.82 | 2.30 | 6.77 | 5.61 | 0.01 | 0.01 | 0.02 | 0.02 | 0.03 | 0.04 | 0.31 | 0.48 | 0.39 | 0.57 | 0.49 | 0.58 | 0.27 | 0.21 | 0.16 |
| **2006** | 1.71 | 1.28 | 3.73 | 3.26 | 8.51 | 6.90 | 1.15 | 0.79 | 3.08 | 2.62 | 7.85 | 6.29 | 0.02 | 0.01 | 0.03 | 0.02 | 0.04 | 0.03 | 0.27 | 0.48 | 0.38 | 0.62 | 0.45 | 0.57 | 0.28 | 0.24 | 0.17 |
| **2007** | 1.74 | 1.28 | 3.77 | 3.18 | 8.68 | 6.88 | 1.17 | 0.82 | 3.09 | 2.60 | 7.90 | 6.24 | 0.02 | 0.01 | 0.02 | 0.02 | 0.01 | 0.03 | 0.28 | 0.45 | 0.38 | 0.56 | 0.54 | 0.61 | 0.27 | 0.27 | 0.23 |
| **2008** | 1.83 | 1.28 | 3.99 | 3.45 | 8.90 | 8.2 | 1.26 | 0.87 | 3.33 | 2.87 | 8.19 | 7.55 | 0.01 | 0.01 | 0.04 | 0.02 | 0.04 | 0.05 | 0.28 | 0.41 | 0.37 | 0.55 | 0.40 | 0.61 | 0.28 | 0.25 | 0.27 |
| **2009** | 1.88 | 1.32 | 4.14 | 3.57 | 9.32 | 8.67 | 1.27 | 0.88 | 3.45 | 2.98 | 8.53 | 8.03 | 0.02 | 0.01 | 0.03 | 0.02 | 0.06 | 0.05 | 0.29 | 0.42 | 0.38 | 0.57 | 0.50 | 0.59 | 0.30 | 0.28 | 0.22 |
| **2010** | 1.90 | 1.42 | 4.31 | 3.74 | 9.42 | 8.95 | 1.29 | 0.95 | 3.58 | 3.12 | 8.68 | 8.31 | 0.03 | 0.01 | 0.03 | 0.02 | 0.03 | 0.04 | 0.25 | 0.45 | 0.37 | 0.60 | 0.46 | 0.60 | 0.33 | 0.32 | 0.25 |
| **2011** | 1.93 | 1.40 | 4.39 | 3.95 | 9.80 | 9.49 | 1.27 | 0.95 | 3.63 | 3.32 | 9.00 | 8.78 | 0.02 | 0.01 | 0.04 | 0.02 | 0.05 | 0.06 | 0.28 | 0.45 | 0.38 | 0.60 | 0.41 | 0.64 | 0.36 | 0.33 | 0.33 |
| **2012** | 2.01 | 1.38 | 4.46 | 3.99 | 9.73 | 9.41 | 1.34 | 0.92 | 3.67 | 3.37 | 8.96 | 8.77 | 0.02 | 0.01 | 0.04 | 0.02 | 0.05 | 0.07 | 0.24 | 0.45 | 0.38 | 0.59 | 0.38 | 0.57 | 0.40 | 0.36 | 0.33 |
| **2013** | 2.16 | 1.45 | 4.68 | 4.23 | 9.43 | 9.41 | 1.46 | 0.98 | 3.87 | 3.55 | 8.60 | 8.77 | 0.01 | 0.01 | 0.03 | 0.03 | 0.06 | 0.05 | 0.28 | 0.46 | 0.37 | 0.64 | 0.38 | 0.60 | 0.41 | 0.41 | 0.39 |
| **2014** | 2.27 | 1.45 | 4.78 | 4.27 | 10.14 | 10.05 | 1.50 | 0.98 | 3.92 | 3.61 | 9.26 | 9.36 | 0.02 | 0.01 | 0.03 | 0.03 | 0.05 | 0.06 | 0.30 | 0.46 | 0.39 | 0.63 | 0.44 | 0.63 | 0.44 | 0.44 | 0.39 |
| **2015** | 2.37 | 1.50 | 5.18 | 4.55 | 11.23 | 11.07 | 1.59 | 1.01 | 4.23 | 3.88 | 10.2 | 10.3 | 0.03 | 0.01 | 0.04 | 0.04 | 0.07 | 0.04 | 0.28 | 0.48 | 0.40 | 0.64 | 0.40 | 0.64 | 0.48 | 0.50 | 0.47 |

W: women; M=men
