# Supplementary material for: Trends in urinary tract infection hospitalization in older adults in Spain from 2000-2015
Source: PLoS One. 2021 Sep 29;16(9):e0257546. doi: 10.1371/journal.pone.0257546 (PMC8480842; doi:10.1371/journal.pone.0257546)
Supplement: S3 File — (DOCX) [file pone.0257546.s003.docx]

|  | APPC | | | JointPoint | APC | | |
| --- | --- | --- | --- | --- | --- | --- | --- |
|  | **APPC** | **CI95%** | **p value** |  | **APC** | **CI95%** | **p value** |
| Non-specified UTI | | | | | | | |
| Total | 4.6 | ( 4.2 ; 5.1 ) | < 0.01 | - | - | - | - |
| Men | 3.9 | ( 2.4 ; 5.5 ) | < 0.01 | 2010; 2013 | 2000-2010 (4.4)  2010-2013 (0.4)  2013-2015 (7.1) | ( 3.6 ; 5.2 )  ( -6.6 ; 7.9 )  ( 0.5 ; 14.2 ) | < 0.01  0.9  < 0.01 |
| Women | 5.4 | (4.5 ; 6.2 ) | < 0.01 | 2009 | 2000-2009 (6.4)  2009-2015 (3.8) | ( 5.1 ; 7.7 )  ( 2.2 ; 5.5 ) | < 0.01  < 0.01 |
| Cystitis | | | | | | | |
| Total | 2.6 | ( -1.5 ; 6.8 ) | 0.2 | 2002 | 2000-2002 (-12.3 )  2002-2015 (5.1) | ( -36.6 ; 21.3 )  ( 3.6 ; 6.6 ) | 0.4  < 0.01 |
| Men | 3.9 | ( 1.7 ; 6.0 ) | < 0.01 | - | - | - | - |
| Women | 2.4 | ( -1.7 ; 6.7 ) | 0.3 | 2002 | 2000-2002 (-13.5)  2002-2015 (5.1) | ( -37.9 ; 20.6 )  (3.5 ; 6.7 ) | 0.4  < 0.01 |
| Pyelonephritis | | | | | | | |
| Total | 0.3 | ( -0.0 ; 0.6 ) | 0.1 | - | - | - | - |
| Men | -0.6 | ( -1.1 ; -0.0 ) | < 0.01 | - | - | - | - |
| Women | 0.6 | ( 0.2 ; 1.0 ) | < 0.01 | - | - | - | - |
| Prostatitis | | | | | | | |
| Total | 6.8 | (6.2 ; 7.3 ) | < 0.01 | 2008 | 2000-2008 (4.9)  2008-2015 (9.0) | (4.0 ; 5.7 )  ( 8.2 ; 9.8 ) | < 0.01  < 0.01) |
| Total UTI | | | | | | | |
| Total | 4.9 | ( 3.2 ; 6.5 ) | < 0.01 | 2010; 2013 | 2000-2010 (5.5)  2010-2013 (1.5)  2013-2015 (6.8) | ( 4.7 ; 6.4 )  ( -6.0 ; 9.6 )  ( -0.3 ; 14.4 ) | < 0.01  0.7  0.1 |
| Men | 3.8 | ( 2.4 ; 5.2 ) | < 0.01 | 2010; 2013 | 2000-2010 (4.0)  2010-2013 (0.9)  2013-2015 (7.1) | ( 3.3 ; 4.7 )  ( -5.3 ; 7.6 )  ( 1.2 ; 13.3 ) | < 0.01  0.7  < 0.01 |
| Women | 4.5 | ( 4.1 ; 4.9 ) | < 0.01 | - |  | - |  |
