# Supplementary material for: Trends in urinary tract infection hospitalization in older adults in Spain from 2000-2015
Source: PLoS One. 2021 Sep 29;16(9):e0257546. doi: 10.1371/journal.pone.0257546 (PMC8480842; doi:10.1371/journal.pone.0257546)
Supplement: S4 File — (DOCX) [file pone.0257546.s004.docx]

|  | **APPC** | | | **JointPoints** | **APC** | | |
| --- | --- | --- | --- | --- | --- | --- | --- |
| **Age** | **APPC** | **IC95%** | **p value** |  | **APC** | **IC95%** | **p value** |
| **Pyelonephritis** | | | | | | | |
| 65-74 | 0.5 | (-1.1 ; 2.0) | 0.6 | 2005; 2008 | 2000-2005 (2.1)  2005-2008 (-4.3)  2008-2015 (1.3) | ( 0.3 ; 4.0 )  (-11.0 ; 3.9 )  ( 0.3 ; 2.4 ) | < 0.01  0.3  < 0.01 |
| 75-84 | 0.6 | ( 0.2 ; 0.9 ) | < 0.01 | - | - | - | - |
| >85 | 0.4 | ( -0.0 ; 0.9) | 0.1 | - | - | - | - |
| **Prostatitis** | | | | | | | |
| 65-74 | 5.5 | (3.8 ; 7.2) | 0.01 | 2003 | 2000-2003 (-0.3)  2003-2015 (7.0) | (-8.5 ; 8.6 )  ( 6.1 ; 7.8 ) | 0.9  < 0.01 |
| 75-84 | 7.7 | ( 6.8 ; 8.7) | 0.01 | - | - | - | - |
| >85 | 7.2 | (3.0 ; 11.6) | < 0.01 | 2004 | 2000-2004 (-3.7)  2004-2015 (11.5) | (-17.9 ; 13.1 )  ( 9.2 ; 13.8 ) | 0.6  < 0.01 |
| **Cystitis** | | | | | | | |
| 65-74 | 3.2 | ( 1.4 ; 5.0 ) | < 0.01 | - | - | - | - |
| 75-84 | 3.9 | ( 2.1 ; 5.6 ) | < 0.01 | - | - | - | - |
| >85 | 4.8 | ( 2.3 ; 7.4 ) | < 0.01 | - | - | - | - |
| **Non-specified UTI** | | | | | | | |
| 65-74 | 3.4 | ( 3.1 ; 3.7 ) | < 0.01 | - | - | - | - |
| 75-84 | 4.5 | ( 4.2 ; 4.9 ) | < 0.01 | - | - | - | - |
| >85 | 5.8 | (3.6 ; 7.9) | < 0.01 | 2010; 2013 | 2000-2010 (6.9  2010-2013 (0.5)  2013-2015 (8.3) | ( 5.7 ; -8.1 )  (-8.9 ; 10.9 )  (-0.7 ; 18.2 ) | < 0.01  0.9  0.1 |
| **Total Urinary Tract Infections** | | | | | | | |
| 65-74 | 2.79 | ( 2.6 ; 3.0) | < 0.01 | - | - | - | - |
| 75-84 | 4.1 | ( 3.8 ; 4.3) | < 0.01 | - | - | - | - |
| >85 | 5.4 | (3.5 ; 7.4) | < 0.01 | 2010; 2013 | 2000-2010 (6.4)  2010-2013 (0.5)  2013-2015 (8.0) | ( 5.4 ; 7.5 )  (-7.9 ; 9.8 )  (-0.1;16.8 ) | < 0.01  0.9  0.1 |
|  |  |  |  |  |  |  |  |
