# Supplementary material for: Trends in urinary tract infection hospitalization in older adults in Spain from 2000-2015
Source: PLoS One. 2021 Sep 29;16(9):e0257546. doi: 10.1371/journal.pone.0257546 (PMC8480842; doi:10.1371/journal.pone.0257546)
Supplement: Attachment — Submitted filename: Response to Reviewers 30_6_21.docx [file pone.0257546.s005.docx]

Dear Editor:

Thank you for the thorough review of our manuscript. All of the comments have been addressed and the manuscript is much improved. Please see our responses to the reviewer’s comments below.

***Editor comments:***

**Thank you for submitting your manuscript entitled "TRENDS IN URINARY TRACT INFECTION HOSPITALIZATION IN OLDER ADULTS IN SPAIN FROM 2000-2015" to PLOS ONE. Your manuscript files have been checked in-house but before we can proceed we need you to address the following issues:

1. We note that you have indicated that data from this study are available upon request. PLOS only allows data to be available upon request if there are legal or ethical restrictions on sharing data publicly. For information on unacceptable data access restrictions, please see http://journals.plos.org/plosone/s/data-availability#loc-unacceptable-data-access-restrictions.


In your revised cover letter, please address the following prompts:


a) If there are ethical or legal restrictions on sharing a de-identified data set, please explain them in detail (e.g., data contain potentially identifying or sensitive patient information) and who has imposed them (e.g., an ethics committee). Please also provide contact information for a data access committee, ethics committee, or other institutional body to which data requests may be sent.**

The study involves the use of patient medical data from The Spanish Centralized Hospital Discharge Database (CMBD). Data are hosted by the Ministry of Health Social Services and Equality (MSSSI). Researchers working in public and private institutions can request the databases by filling, signing and sending a questionnaire available at the MSSSI website. In this questionnaire a signed Confidentiality Commitment is required. All data are anonymized and de-identified by the MSSSI before it is provided to applicants. According to this Confidentiality Commitment signed with the MSSSI, researchers cannot provide the data to other researchers that must request the data directly to the MSSSI. Data can be obtained through the following link: <http://www.msssi.gob.es/estadEstudios/estadisticas/estadisticas/estMinisterio/SolicitudCMBDdocs/Formulario_Peticion_Datos_CMBD.pdf>.

**2. Please amend your list of authors on the manuscript to ensure that each author is linked to an affiliation.**

**We note that you have included affiliation numbers 1-10 however only affiliations 1-9 have authors linked to them. Please amend affiliation 10 to link an author to it or remove if added in error.**

The proposed changes have been made. Thank you.

**3. Please include a separate legend for each figure in your manuscript.**

We believe we have made the requested changes. Thank you

**4. Thank you for including your ethics statement on the online submission form:  "The study was approved by the Hospital Fundación Alcorcon Ethics Research Committee (20/125) favorably evaluated by the Central Research Commission of the Primary Healthcare Management of Madrid (57/20). The need for consent was waived by the Ethics Committee (20/125). The CMBD data were fully anonymized by the Ministry of Health before investigators accessed to them.".  

To help ensure that the wording of your manuscript is suitable for publication, would you please also add this statement at the beginning of the Methods section of your manuscript file.**

We have included it in the Ethics Statement section, replacing the previous incomplete text. Thanks for the recommendation

Yours faithfully,

Ricardo Rodriguez
