# Supplementary material for: Trends in urinary tract infection hospitalization in older adults in Spain from 2000-2015
Source: PLoS One. 2021 Sep 29;16(9):e0257546. doi: 10.1371/journal.pone.0257546 (PMC8480842; doi:10.1371/journal.pone.0257546)
Supplement: Attachment — Submitted filename: Response to Reviewers03_09_21.docx [file pone.0257546.s007.docx]

Dear Editor:

Thanks for your review. See our responses to reviewers' comments below.

***Editor comments:***

There are sentences in Spanish in the text that require translation to English.

1. **"Análisis de tendencias (joint-point) global, por tipo de itu, por sexo y por grupo de edad" "Por tipo de ITU"**

Thank you very much. We have replaced "Análisis de tendencias (joint-point) global, por tipo de itu, por sexo y por grupo de edad" and "Por tipo de ITU" with “Analysis of trends (joinpoint) globally and by UTI type, sex, and age group” and “By UTI type

Yours faithfully,

Ricardo Rodriguez
